# Comparison of muscle activation and proprioception during landing at different angles between individuals with chronic ankle instability and healthy controls

**DOI:** 10.1186/s12891-025-09212-8

**Published:** 2025-10-08

**Authors:** Serkan Uzlasir, Beyza Tayfur, Erhan Işıkdemir, Abdulhamit Tayfur

**Affiliations:** 1 Faculty of Sports Sciences, Nevşehir Hacı Bektaş Veli University, Nevşehir, Türkiye; 2 School of Physical Therapy and Rehabilitation, Ahi Evran University, Kırşehir, Türkiye

**Keywords:** Ankle sprains, Electromyography, Proprioception, Joint instability

## Abstract

**Background:**

Chronic Ankle Instability (CAI) is associated with proprioceptive deficits and altered neuromuscular control, yet limited studies have examined these factors concurrently during functional tasks like jump-landing.

**Methods:**

A total of 44 participants, including 21 healthy individuals and 23 with CAI, participated in a crossover experimental study. An EMG system (Noraxon, USA) was used bilaterally to assess muscle activation of the key muscles involved in ankle movement during jump-landing activities. Specifically, medial gastrocnemius (MG) and tibialis anterior (TA) muscles were evaluated. To identify differences in The Ankle Inversion Discrimination Apparatus for Landing (AIDAL) parameters between groups, an independent samples t-test was conducted. Linear mixed modeling with repeated measures was performed to analyze muscle activation differences during jump-landing at different angles.

**Results:**

Individuals with CAI exhibited significantly lower AIDAL slope estimates compared to healthy controls, with a moderate effect size (*p* < 0.001, d = 0.78). In the EMG results, no statistically significant difference was observed between the CAI group and healthy controls (*p* = 0.431) for TA and (*p* = 0.699) for MG. Statistically significant differences were found in the activation of the MG muscle between 10^o^ and 14^o^ (*p* = 0.009), between 12^o^ and 16^o^ (*p* = 0.041) and between 14^o^ and 16^o^ (*p* = 0.005).

**Conclusion:**

This study indicates that individuals with CAI experience impaired proprioception compared to healthy individuals, while showing no notable differences in key muscle activation patterns. It highlights the necessity of rehabilitation strategies focused on proprioception.

## Background

Lateral ankle sprains are the most common musculoskeletal injuries, accounting for 15% of all injuries in sports [1]. More than 40% of individuals with lateral ankle sprains develop chronic ankle instability (CAI) [2]. CAI is characterized by repetitive episodes or perceptions of the ankle giving way; ongoing symptoms Like pain, weakness, or reduced ankle range of motion; diminished self-reported function; and recurrent ankle sprains for more than 1 year after injury [3]. While healthy individuals can successfully compensate for the alterations of somatosensory inputs around foot-ankle by reweighting other sensory inputs, this ability is reduced in individuals with CAI [4, 5]. Altered motor planning, execution and sensory processing may represent mechanisms underlying impairments in people with CAI [6]. Persistent spinal and supraspinal changes result in inconsistent movement control [7]. Therefore, it is important to evaluate the proprioceptive inputs.

Proprioception is the afferent information derived from sensory receptors necessary for the control of movement [8]. The foot-ankle complex is the only part of the body in contact with the ground during most sports activities and provides information to adjust ankle positions and upper body movements to successfully perform complex motor tasks of elite sports [9, 10]. Damage to peripheral mechanoreceptors providing proprioceptive input may lead to alterations in neuromuscular control [11]. This can disrupt proprioceptive afferent, causing changes in sensory organization strategies over time [12, 13]. Moreover, individuals with CAI demonstrate sensorimotor insufficiencies and proprioception deficits [14, 15], as well as biomechanical variations in lower limb movement patterns, motor strategies, muscle activation, and leg stiffness control during landing [16–20].

In individuals with ankle instability, increased peroneus longus (PL) activity during landing from a jump [21], and similarly, increased tibialis anterior (TA) activity during walking [22] have been observed compared to healthy controls. Individuals with CAI also have abnormal activation of the peroneal and TA muscles [23]. Significant increases in PL and TA muscle activity were found just before landing, and ankle evertor movements were increased when landing in different directions [24]. In addition, during walking, the activation of the TA was found to be lower, whereas the activation of the MG was higher [25]. In this case, increases and decreases in magnitude have been reported in the same muscles during similar tasks.

It has been reported that individuals with CAI demonstrate increased frontal displacement and limited sagittal displacement [26]. However, during jogging, a reduction in ankle dorsiflexion ranging from 9% to 25% of the gait cycle (Initial contact to terminal swing) has been observed compared to controls [27]. Furthermore, individuals with CAI exhibit reduced ankle plantarflexion [28, 29] and dorsiflexion during landing [30]. During the same time period, compared to the control group, they performed dorsiflexion at a slower rate, resulting in decreased sagittal plane angular velocity [30]. Consequently, this condition was found to cause a reduction in eccentric power generation in the sagittal plane [30]. Tight calf musculature may be a factor contributing to this finding. Therefore, during inversion landing, the activation of the TA and MG muscles, which act antagonistically in the sagittal plane, is of great importance.

Evaluating both muscle activation and proprioception during landing, which is a part of daily life, is valuable for obtaining simultaneous neuromuscular information in CAI. The lack of studies with comprehensive comparisons and test methodologies may be the primary reason for inconsistent findings [31]. We aimed to evaluate muscle activation and proprioception concurrently to identify neuromuscular deficits in individuals with CAI. Consequently, our hypothesis was that proprioception and muscle activation in individuals with CAI differ from healthy individuals during jump-landing at different angles.

## Methods

### Participants

A total of 126 volunteers were screened for enrollment. CAI was defined as individuals who: have sustained at least two lateral ankle sprains; have experienced at least one episode of giving way within the past 6-months; have unilateral CAI; have a score of ≥ 11 in IdFAI as per the International Ankle Consortium’s statement [32]. Turkish version of IdFAI questionnaire were administered [33, 34].

A total of 44 participants, 21 healthy (20.4±0.3 years) and 23 with CAI (21.3±0.6 years), participated in a crossover experimental study (Table 1). Exclusion criteria for CAI were: <18 or > 35 years of age, vestibular and vision problems, acute lower extremity or head injuries (< 6 weeks), chronic musculoskeletal conditions known to affect balance (e.g., ACL deficiency), history of ankle surgeries, and acute injuries that may impact balance, sensitivity to strobe Lighting, being pregnant, having diabetes, and vertigo. The sample size was determined based on an effect size of 0.85, 5% type I error, and 80% power [35]. This power analysis is based on a study that includes lower limb muscle activity values during vertical jumping in both the CAI and control groups [35]. Power analysis was conducted using the G-Power (version 3.1.9.3, Heinrich-Heine-Universität Düsseldorf, Germany). Two healthy participants were excluded due to technical issues with electromyography (EMG) data, resulting in 21 healthy participants. The study was conducted in the university laboratory with a total of 44 participants. Subjects read and signed an informed consent form approved by Nevşehir Haci Bektaş Veli University’s Non-interventional Clinical Research Ethics Board (Number: 2023.04.01) prior to participation.

**Table 1:** Demographics of participants SD: standard deviation CAI: chronic ankle instability, CON: control, idfai: identification of functional ankle instability questionnaire

Sex (Male/Female)	CAI (*n* = 23) 6/17	CON (*n* = 21) 7/14	
	**Mean ± SD**	**Mean ± SD**	***P*** **value**
Age (years)	21.26±2.64	20.42±1.50	0.114
Height (m)	1.65±0.91	1.66±0.90	0.988
Mass (kg)	61.90±10.06	62.17±13.18	0.252
IdFAI	16.30±3.95	0,63 ± 1.25	< 0.001

### Procedures

An EMG system (Noraxon USA) was used bilaterally to assess activation of medial gastrocnemius (MG) and TA muscles. Electrode placement followed the SENIAM Guidelines [36]. Prior to placing the EMG sensors, the skin was prepared by cleaning with alcohol, gentle abrasion, and shaving to optimize signal quality. Four surface electrodes were placed on each participant, using bipolar Ag/AgCl electrodes with an inter-electrode distance of 2 cm. The electrode dimensions were 1 cm in width, with a common mode rejection ratio of > 80 dB and an input impedance greater than 10 mΩ. The EMG data were sampled at a frequency of 2000 Hz.

**Fig. 1:**
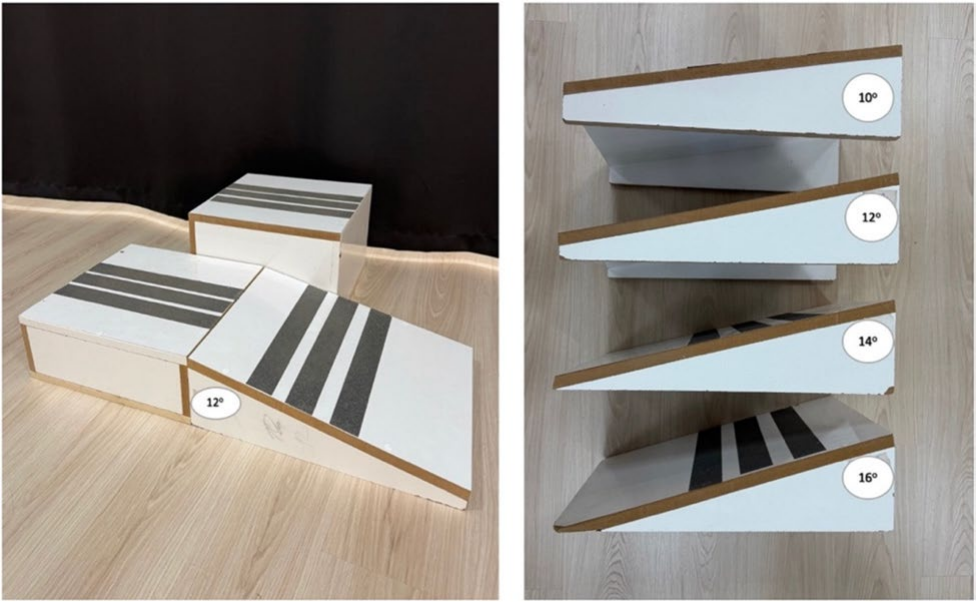
The ankle inversion discrimination apparatus for landing

The Ankle Inversion Discrimination Apparatus for Landing (AIDAL) was used to evaluate proprioceptive discrimination of ankle inversion during landing [37]. The AIDAL demonstrated good test–retest reliability in both CAI and non-CAI groups, suggesting that measuring ankle inversion proprioception during landing may be a valuable tool for evaluating rehabilitation outcomes in individuals with CAI [37]. The AIDAL is specifically designed to assess an individual’s capacity to utilize proprioceptive input in distinguishing between varying degrees of ankle inversion upon landing. The apparatus comprises three components: the take-off platform (Fig. 1), a horizontal landing platform for the support leg (Fig. 1), and an inclined landing platform for the test leg (Fig. 1). The height differential between platforms A and B is 10 cm. The inclined platform allows for four possible ankle inversion angles as 10°, 12°, 14°, and 16° (Fig. 1).

The experiments were conducted in the Sports Sciences Laboratory at Nevşehir Hacı Bektaş Veli University. Participants underwent a jump test and were instructed to keep their heads and eyes facing forward, avoiding visual information from the apparatus to prevent any influence on the landing platform perception. For each trial, participants stepped onto the AIDAL from the take-off platform, advanced with the test foot, and landed simultaneously on both feet with their hips and knees naturally flexed. We evaluated the post-landing phase. A 1080p camera was synchronized with the EMG system (Noraxon, USA). The moment of full foot contact was considered as the point of landing. The testing procedure consisted of three rounds of familiarization of the four ankle inversion landing positions (12 trials) presented in order, and 40 trials of testing (10 for each of the ankle inversion) presented in random order [37]. Although fatigue may influence neuromuscular performance, participants completed only 40 trials without rest, consistent with previous AIDAL studies [37, 38]. Therefore, the potential effect of fatigue on our findings is Likely minimal but should be acknowledged as a Limitation. Participants were required to recall the four distinct ankle inversion positions learned during the practice session and make a judgment on the degree of ankle inversion for each test trial immediately after landing. No feedback was provided regarding the accuracy of their judgments. Participants received one point for each correct guess across a total of 40 jumps, resulting in a maximum possible score of 40 points.

### Data processing

**Fig. 2:**
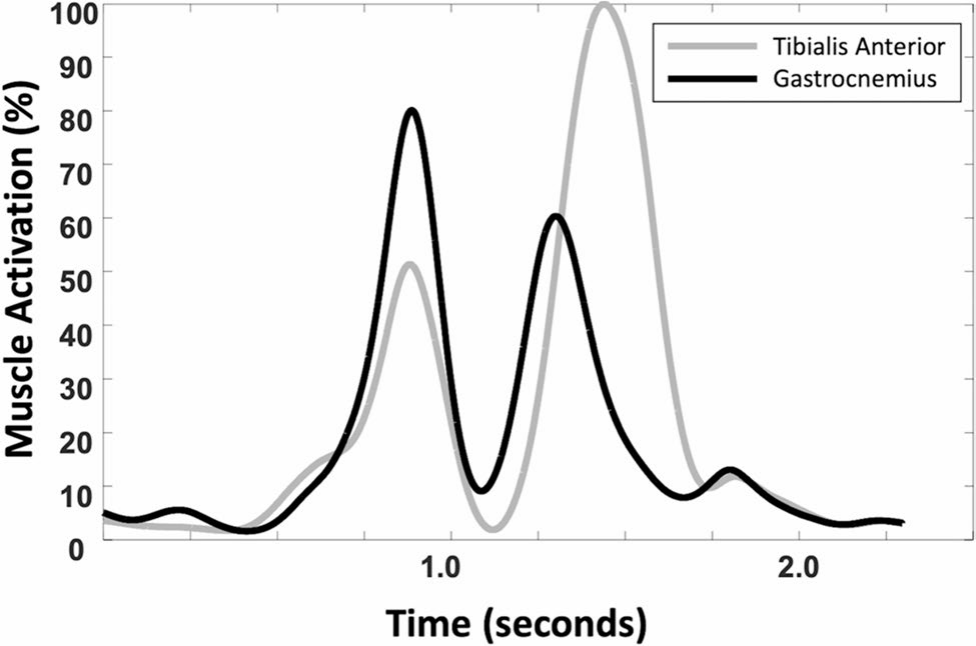
EMG activity of the tibialis anterior and medial gastrocnemius muscles

All EMG data were processed offline with a custom-built MATLAB script (MATLAB version R2021b; MathWorks). EMG signals were bandpass filtered using a fourth-order, zero-lag Butterworth filter with cutoff frequencies of 20 Hz to 450 Hz to remove any movement artifacts. The signals then full-wave rectified and normalized to the peak muscle EMG amplitude obtained during the jump-landing activity for each respective muscle. Peak amplitude of the percentage EMGs were used to represent the activity of each muscle. The EMG signals were averaged over multiple trials. The EMG activity of the TA and MG muscles is presented in Fig. 2.

### Statistical analysis

Data analysis was performed using SPSS software version 25.0 (IBM Corp, Armonk, NY). All data were inspected form normality using both graphical (histograms, probability plots) and analytical (Shapiro-Wilk test) methods. To identify differences in AIDAL parameters between groups, an independent samples t-test was conducted. Cohen’s d effect size was calculated. An effect size of < 0.2 was considered negligible, between 0.2 and 0.5 as small, between 0.5 and 0.8 as moderate, and > 0.8 as large [39]. Linear mixed modeling with repeated measures was performed to analyze muscle activation differences during jump-landing at different angles. Group (healthy and CAI) X landing angles (10-12-14-16 degrees) interaction were checked for each muscle (TA and MG) activation. Group and landing angles were treated as fixed effects and participant as a random effect. When variables included in the model were significant, post hoc analyses were performed using main effects comparison with Sidak. Statistical significance was set at *p* < 0.05.

## Results

### AIDAL (The ankle inversion discrimination apparatus for Landing)

Individuals with CAI had a mean score of 21.90 ± 1.20 while healthy individuals had 27.14± 1.33. Individuals with CAI exhibited significantly lower slope estimates compared to healthy controls, with a moderate effect size (*p* < 0.001, d = 0.78), (Table 2).

**Table 2:** AIDAL scores

	CAI Mean ± SD	CON Mean ± SD	*P* Value Mean ± SD
**AIDAL (scores)**	21.90 ± 1.20	27.14± 1.33	**0.001**

*AIDAL* The Ankle Inversion Discrimination Apparatus for Landing, *CAI* Chronic Ankle Instability, *CON* Control

### Muscle activation comparisons between groups

Tibialis anterior peak muscle activation was found to be 47.62 (95% CI 41.62–53.63) in individuals with CAI and 44.19 (95% CI 37.91–50.48) in healthy individuals. No statistically significant difference was observed between the CAI group and healthy controls (*p* = 0.431) for TA, as shown in Table 3.

**Table 3:** Muscle activation between groups

Muscles (percentage)	Group	Mean ± SD (95% CI)	*P* value
**Tibialis Anterior**	*CAI* (*n* = 23)	47.62± 14.49 (41.62–53.63)	0.431
	**CON** (*n* = 21)	44.19± 15.52 (37.91–50.48)	
**Medial Head of Gastrocnemius**	**CAI** (*n* = 23)	53.39± 15.59 (47.92–58.86)	0.699
	**CON** (*n* = 21)	54.88± 9.93 (47.92–58.86)	

*CAI* Chronic Ankle Instability,*CON* Control,*CI* Confidence Interval

Peak muscle activation of the medial head of gastrocnemius was 53.39 (95% CI 47.92–58.86) in CAI and 54.87 (95% CI 49.41–60.35) in healthy group. No statistically significant difference was found between groups (*p* = 0.699) for MG, as presented in Table 3.

### Muscle activation at different slope angles

Muscle activation of the tibialis anterior and gastrocnemius muscles at different slopes is given in Table 4. There is no statistically significant difference in TA activation in different slopes. (*p* > 0.05). Statistically significant differences were found in the activation of the gastrocnemius medialis muscle between 10^o^ and 14^o^ (*p* = 0.009), between 12^o^ and 16^o^ (*p* = 0.041) and between 14^o^ and 16^o^ (*p* = 0.005).

**Table 4:** Muscle activation at different slope angles

Muscles (percentage)	Group	Mean ± SD			
		Slope 10 ^o^	Slope 12 ^o^	Slope 14 ^o^	Slope 16 ^o^
**Tibialis Anterior**	**CAI** (*n* = 23)	48.95±14.23	46.22±14.30	47.85±16.06	47.47±14.13
	**CON** (*n* = 21)	45.43±15.28	43.03±15.81	43.25±16.01	45.08±15.97
**Medial Head of Gastrocnemius**	**CAI** (*n* = 23)	56.43±15.46(*)	51.93±13.77**(**^**†**^)	50.34±17.27(*,^**§**^)	54.86±16.07(^**†,§**^)
	**CON** (*n* = 21)	54.96±8.71(*)	53.76±9.49(^**†**^)	53.85±11.02 (*,^**§**^)	56.96±10.71 (^**†,§**^)

*CAI* Chronic Ankle Instability, *CON* Control, *CI* Confidence Interval, ^*^ Significant Difference Between 10 ^o^ and 14 ^o^ (*p* < 0.05), ^**†**^ Significant Difference Between 12 ^o^ and 16 ^o^ (*p* < 0.05), ^**§**^ Significant Difference Between 14 ^o^ and 16 ^o^ (*p* < 0.05)

## Discussion

In this study, the effects of CAI on proprioception and muscle activation during jump-landing on inclined slopes were examined. Significantly worse proprioception were found for individuals with CAI compared to healthy individuals. However, no differences were observed in TA and MG muscle activations between groups. Additionally, differences were found in the activation of the MG muscle between different angles.

The significant decline in proprioception among individuals with CAI is likely a result of damage to the somatosensory receptors. Healthy individuals can successfully compensate for the alteration of somatosensory inputs around the foot-ankle complex by reweighting other sensory inputs; however, this compensatory ability is reduced in CAI [4, 5]. Individuals with CAI often exhibit perceptual, pathomechanical, and perceptual-motor deficits, such as decreased postural control, muscle weakness, pathological laxity, and diminished somatosensation [3, 40]. Somatosensory impairment, has significant consequences for postural control, a key factor in preventing recurrent ankle injuries [40]. In support, our study shows individuals with CAI may not accurately estimate slopes, revealing a considerable extent of somatosensory impairment.

Alterations in motor planning, execution, and sensory processing may represent underlying mechanisms for one of the most commonly described impairments in CAI [6]. For instance, anesthetizing the lateral ankle ligaments in healthy individuals does not impair their ability to maintain upright stance control [5]. Likewise, disrupting afferent signals from the plantar surface does not negatively affect stance control in healthy individuals [41]. These findings suggest that, in healthy adults, the removal of somatosensory inputs may be compensated for by dynamically reweighting other sensory receptors (e.g., visual receptors, sensory organization strategies). In the present study, the proprioceptive deterioration observed in individuals with CAI, likely due to mechanoreceptor damage, highlights the importance of proprioceptive training and rehabilitation. Proprioceptive exercises should target both central and peripheral stimuli, incorporating visual, vestibular, and somatosensory inputs—key components of the postural mechanism. Given the potential for both peripheral and central changes associated with CAI [11, 42–44], rehabilitation strategies must reflect these complexities.

In this study the AIDAL was preferred to assess ankle proprioception as the joint position reproduction (JPR) method or the passive motion detection threshold method (TTDPM), fails to detect any proprioceptive changes in individuals with CAI [45]. A recent systematic review and meta-analysis showed the limited sensitivity of JPR and passive motion detection threshold methods in detecting proprioceptive deficits in CAI [46], suggesting that the JPR and TTDPM methods lack ecological validity because they do not simulate the actions that occur during ankle injury [46]. To address this limitation, the AIDAL assessment parameters were chosen in this study as they more closely replicate the sensory-rich environment typical of sports activities [47, 48]. We found no differences in the TA or MG muscle activation between healthy and CAI groups. Additionally, there were no differences in tibialis anterior activation, but significant differences for the MG muscle at different slopes. Perceptual dysfunction, a phenomenon closely associated with physiological changes resulting from ligament injuries in individuals with CAI [46, 49] may contribute to these findings. Ligament injuries are linked to decreased gamma motor neuron activation and reduced sensitivity of muscle spindles, which impairs the individual’s ability to perceive mechanical stimuli (e.g., force and vibration). They also affect proprioceptive feedback regarding joint position and movement, ultimately compromising ankle stability [50, 51]. Reduced ankle stabilization due to compromised proprioceptive feedback may have influenced muscle activation. A second factor may be reduced knee flexion angle during landing following a jump in individuals with CAI [52, 53]. Reduced knee flexion may limit the activation of the proximal head of the biarticular medial gastrocnemius, which in turn could affect the function of the tibialis anterior that operates eccentrically and concentrically. As a result, it is thought that this condition may restrict the optimal activation of both muscles. Gribble et al. observed decreased knee flexion angle during landing prior to contact in patients with CAI [54]. Similarly, a reduction in knee cushioning capacity and a significant decrease in knee flexion angle at peak anterior tibial shear force were seen in patients with CAI [55].

Individuals with CAI demonstrate specific adaptive kinematic changes compared to healthy populations. They typically exhibit a smaller ankle plantarflexion angle and increased hip flexion at initial contact during landing [28, 29], which results in greater forward trunk rotation and a shift in the center of gravity. Additionally, previous studies have reported reductions in both ankle plantarflexion and dorsiflexion in individuals with CAI during landing tasks [28–30]. These restrictions in joint range of motion lead to slower dorsiflexion rates and reduced sagittal plane angular velocity compared to healthy individuals. As a consequence, there is a notable decrease in eccentric power generation in the sagittal plane. This reduced capacity for energy absorption may impair dynamic joint stability during landing. In this context, the role of TA and MG muscles—working as antagonists in the sagittal plane—becomes critically important. Although no significant differences were observed in peak EMG activation levels of TA and MG between groups, this may reflect a compensatory mechanism in individuals with CAI aimed at maintaining stability under biomechanically compromised conditions. The limited ankle range of motion may have constrained the ability of these muscles to adjust activation levels dynamically, reducing their eccentric force contribution during impact absorption. Taken together, these findings suggest that CAI may function as a reflexive, automatic protective response to repeated ankle trauma. One of the key objectives of our study was to explore the existence of this compensatory strategy, and our data provide evidence in support of this hypothesis.

The absence of a single-foot landing on the AIDAL platform, combined with the bilateral jump from the platform and the flat, unaffected landing surface, may have allowed the unaffected side to compensate for the CAI side by landing earlier. Landing from different heights and in various directions can result in different muscle activation patterns. Single-foot landings pose the greatest risk for ankle sprains [56]. There are alterations in kinematics, kinetics, dynamic postural stability, and muscle activity during landing tasks in individuals with CAI [57, 58]. However, most studies have focused on forward unilateral landings, which do not fully replicate the multidirectional jumps and landings typically encountered in real sports scenarios. In our study, we focused on a forward landing technique to simultaneously assess muscle activation and proprioception. While this simultaneous measurement approach offers certain advantages, it may also have limitations. As noted by Wikstrom et al. [59], relying solely on forward landing tasks may not provide sufficient predictive power when assessing the risk of lower extremity injuries. A recent study by Sinsurin et al. [24] reported significant increases in peroneus longus and tibialis anterior muscle activity just prior to landing, along with increased ankle evertor movements during forward, diagonal, and lateral landings. However, the AIDAL method is designed to assess only anterior jumps and does not accommodate multidirectional jumping. Consequently, the anticipated activation differences, particularly in the tibialis anterior muscle, may not have been observed, potentially due to reduced plantar flexion stabilization. Another aspect supporting this theory is the activation differences observed at varying slope levels. When comparing the slope levels, no significant differences were observed in tibialis anterior activation; however, a significant difference was found in MG activation. The reduction in plantar flexion and the consequent compensatory mechanisms did not result in significant activation differences in the TA, even at different slopes. However, the observed difference in gastrocnemius activation may be attributed to the tendency for ankle inversion during descent, potentially influenced by the incline of the ramp.

The absence of differences in muscle activation despite variations in proprioception is particularly noteworthy. The precise neurophysiological mechanisms underlying CAI remain unclear, as CAI is associated with both mechanical factors (e.g., ligamentous laxity) and perceptual instability (e.g., sensorimotor deficits) [60]. Assessing somatosensory differences alongside mechanical damage may provide valuable insights into better understanding the underlying mechanisms. Our study simultaneously evaluated two distinct neurophysiological pathways. While sensorimotor differences in CAI can be detected using AIDAL, additional strategies are needed to assess mechanical alterations. Future research evaluating muscle activation in the knee, hip, and back during multidirectional jumps, where ankle angles are more clearly defined, may provide further insights into the broader effects of CAI on the body.

This study has several limitations that should be considered. First, the use of only forward (anterior) landing tasks Limits the ecological validity of the findings, as multidirectional landings are more representative of real sports scenarios. Second, the bilateral landing approach may have allowed compensation by the unaffected Limb, potentially masking neuromuscular deficits in the CAI limb. Third, the EMG analysis was limited to the TA and medial MG; inclusion of other key stabilizers, such as the peroneus longus, soleus, and gluteal muscles, could have provided a more comprehensive understanding. Fourth, participants performed 40 consecutive jumps without rest; although this protocol is consistent with previous studies [37, 38], the possible influence of fatigue on neuromuscular responses cannot be completely ruled out. Future studies should address these limitations by incorporating multidirectional tasks, a broader muscle set, and advanced biomechanical analyses.

## Conclusions

This study highlights impaired proprioception in individuals with CAI, while muscle activation patterns of the tibialis anterior and medial gastrocnemius showed no notable differences. These findings suggest that proprioception-focused rehabilitation protocols are essential, particularly under dynamic landing conditions. Future research should address multidirectional tasks to further clarify sensorimotor adaptations in CAI.

## Data Availability

The datasets analyzed during the current study are available from the corresponding author on reasonable request.

## Abbreviations

CAI: Chronic Ankle Instability
MG: Medial gastrocnemius
TA: Tibialis anterior
AIDAL: The Ankle Inversion Discrimination Apparatus for Landing
EMG: Electromyography
JPR: Joint Position Reproduction
TTDPM: Passive motion detection threshold method
